# Radiologic Hallmarks of Colonic Volvulus: A Narrative Review

**DOI:** 10.7759/cureus.105186

**Published:** 2026-03-13

**Authors:** Giva Mariel López Saborio, Esteban Alvarado Sanabria, María Alejandra Villalobos de Ycaza, Jean Jaasiel Mora Murillo, Mónica Quirós Alvarado, Kendal Leandro Sandí

**Affiliations:** 1 Facultad de Medicina, Universidad de Costa Rica, San José, CRI

**Keywords:** bird's beak sign, coffee-bean sign, ileosigmoid knot, radiologic diagnosis of volvulus, the frimann dahl sign, the liver overlap sign, the northern exposure sign, transition zone, volvulus, whirl sign

## Abstract

Volvulus is a cause of acute abdominal pain and imaging methods are essential for the diagnosis. During basic medical training, it is taught that the coffee bean sign, visible on radiographs, is indicative of an intestinal volvulus; however, numerous radiological signs are also visible on X-ray, fluoroscopy, CT, and MRI. Therefore, a literature review was conducted using ScienceDirect, Elsevier, PubMed Central, and Ovid Lippincott Williams & Wilkins databases, from which 32 articles in English and Spanish were extracted. This review found that there are many more signs for diagnosing colonic volvulus than just the coffee bean. CT is the most sensitive and specific method; if X-ray is combined with contrast enema, the sensitivity increases. The preferred methods for people who can't be exposed to ionizing radiation are US and MRI.

## Introduction and background

The term *volvulus *comes from the Latin word "volvere", meaning to twist. In medicine, it refers to a pathological twisting of a section of the gastrointestinal tract [1]. The digestive tract is a dynamic system of organs, so twisting of the intestinal loops less than 180° is physiological and usually resolves spontaneously, while rotations greater than 180° generally obstruct intestinal passage, leading to volvulus. Rotations greater than 360° compromise the vascular structures, leading to ischemia and necrosis [2]. After obstruction develops, peristalsis continues in an attempt to propel intestinal contents through the bowel. Consequently, bowel loops proximal to the obstruction become progressively dilated with swallowed air, whereas distal loops eventually decompress as their contents are evacuated. Understanding the distribution of gas in bowel obstruction is fundamental for recognizing imaging findings [3].

The most widely recognized radiologic sign used to diagnose volvulus is the coffee bean sign, visible on plain abdominal radiography and computed tomography (CT). It is also named the omega sign, horseshoe sign, inverted U sign, and bent inner tube sign [4]. It was first described by Leo Rigler, one of the giants of gastrointestinal radiology, in a 1944 review. He described that in patients with strangulating bowel obstruction, the gas-filled bowel looked like a "coffee bean”, with two loops separated by a wide band of increased density [5]. However, several additional radiologic signs may assist in establishing the diagnosis, particularly in patients whose imaging findings do not present with the classic appearance. Before evaluating imaging findings, it is important to understand the epidemiology, risk factors, and clinical presentation of colonic volvulus in order to appropriately suspect the condition and guide management.

Colonic volvulus is the third most common cause of large bowel obstruction, after malignant neoplasms and diverticular strictures. In the United States and Western Europe, colonic volvulus accounts for approximately 10-15% of large bowel obstructions. In contrast, the condition is more prevalent in certain regions of the world where sigmoid volvulus represents 60-70% of large bowel obstruction cases and cecal volvulus accounts for 25-40% of cases. These regions - including Africa, the Middle East, and South America - are collectively referred to as the “volvulus belt” [6,7]. In Western countries, sigmoid volvulus most commonly affects men older than 70 years. In contrast, in the volvulus belt it usually affects younger men, often beginning in the fourth decade of life. Cecal volvulus tends to occur in younger women, typically under 60 years of age [7,8].

The anatomic site where it occurs most frequently is the sigmoid colon, followed by the cecum; other types of volvulus are less common. According to the mechanism of torsion, the subtypes are organo-axial or mesentero-axial volvulus [6,8]. Risk factors for developing a sigmoid or cecal volvulus include elongated colon, chronic constipation, frequent laxative use, neuropsychiatric disorders, prolonged bed rest, previous abdominal surgery, Chagas disease (especially in South America), and diabetes [1,6,9]. Additional factors, such as overeating after prolonged fasting, may precipitate volvulus because the rapid transport of bulky contents can promote torsion of the sigmoid colon. Similarly, a high-fiber diet may increase stool volume and fecal load, raising intraluminal pressure and impairing elastogenesis of the colonic wall, eventually resulting in an elongated colon (dolichosigmoid). Living at altitudes above 1500 meters has also been associated with increased incidence due to expansion of intraluminal gases caused by lower atmospheric pressure [2].

In contrast to adult epidemiology, pediatric cases of volvulus are reported more frequently in Western countries, including North America. The main risk factors in children include a long mesentery and Hirschsprung’s disease, particularly when diagnosis is delayed beyond infancy [10,11]. Other risk factors include neurodevelopmental delay, anal stenosis, constipation, and prune belly syndrome [12]. Nevertheless, volvulus remains a rare condition in the pediatric population [10,11].

In patients with suspected volvulus, initial evaluation should include a focused history, physical examination, and basic laboratory assessment. Regardless of type or location, hemodynamic stability and peritonitis should be assessed initially [6-8]. The clinical manifestations are those expected for intestinal obstruction, such as acute abdominal pain, abdominal distention, nausea, and vomiting. The onset of symptoms may vary depending on the location of the volvulus. In sigmoid volvulus, symptoms often develop gradually over two to five days, whereas in cecal volvulus the onset is typically more abrupt and may occur within hours [6]. Plain abdominal radiography is usually the initial imaging modality. When radiographic findings are inconclusive, CT imaging - with or without contrast - can provide additional diagnostic information. If the diagnosis remains uncertain despite cross-sectional imaging, a water-soluble contrast enema may help confirm the diagnosis. Once the diagnosis is established, endoscopic detorsion is typically the initial treatment for sigmoid volvulus. Surgical resection may be required when endoscopic detorsion fails, in cases of recurrence, or as first-line treatment for cecal volvulus [7,8].

## Review

Methods

A narrative literature review was conducted to identify and summarize radiologic signs, other than the classic coffee bean sign on plain radiography, that may assist in the diagnosis of colonic volvulus. A comprehensive literature search was performed between January and February 2026 using the following electronic databases: ScienceDirect, PubMed Central, and the Ovid Lippincott Williams & Wilkins Total Access Collection. Additional relevant publications available through Elsevier platforms were also reviewed. The search strategy included combinations of the following keywords: “volvulus,” “colonic volvulus,” “diagnosis of volvulus,” “coffee bean sign,” “whirl sign,” “computed tomography volvulus,” “CT volvulus,” “ultrasound volvulus,” “fluoroscopy volvulus,” and “MRI volvulus.” Boolean operators (AND/OR) were used to refine the search results. Medical Subject Headings (MeSH) terms were not used. Articles published in English or Spanish within the last 10 years were primarily considered. However, older publications were also included when they contained historically relevant or frequently cited descriptions of radiologic signs associated with colonic volvulus. No restrictions were applied regarding study design; therefore, different types of publications were included, such as literature reviews, systematic reviews, case reports, and observational studies describing imaging findings of colonic volvulus. Studies focusing primarily on the treatment of volvulus or addressing midgut volvulus were excluded to maintain focus on diagnostic imaging findings related to colonic volvulus. The selection process involved an initial screening of titles and abstracts to assess relevance. Articles meeting the inclusion criteria underwent full-text review. A total of 41 articles were initially identified. After removal of duplicate records, 39 articles remained for screening. Following application of the eligibility criteria, 32 articles were included in the final review.

Findings on X-ray

Radiography is a rapid, widely available, and well-tolerated tool in unstable patients or those with peritonitis, making it invaluable in the initial management of abdominal pain. In adults, it has a sensitivity of 26%-42% for colonic volvulus in general, but it is more useful for diagnosing sigmoid volvulus than cecal volvulus or those situated in other zones of the colon [6,7,13]. 

In sigmoid volvulus, there are many features to look for in X-rays: The sigmoid colon dilates disproportionately when compared to the proximal colonic segments, gas is not seen in the rectum due to the proximal obstruction, and four different signs have been described, which are: the coffee bean sign, the northern exposure sign, the Frimann-Dahl sign, and the liver overlap sign; an explanation of each one is available on Table 1 [4,6,7,14-17].

**Table 1:** Main signs of sigmoid volvulus on radiography

Sign	Description
The coffee bean sign	The apposition of the medial walls of the dilated bowel forms the cleft of the coffee bean; lateral walls of the dilated bowel loops form the outer wall of the bean. This sign can also be seen in cecal volvulus, by CT and MRI [4,6,7].
The northern exposure sign	A dilated sigmoid colon that repositions from the pelvis to extend above the transverse colon (at the midline or in the left subdiaphragmatic region). The image is obtained with the patient supine [4,14,15]. This feature indicates sigmoid volvulus with 86% sensitivity and 100% specificity [16,17].
The Frimann Dahl sign	At the site of obstruction, three dense lines can be seen converging. The two lateral lines represent the lateral walls of the dilated bowel and the middle line by apposition of the medial walls of the bowel loops [4].
The liver overlap sign	The sigmoid loop is seen projecting to the right upper quadrant, projecting over the liver shadow; this finding is highly specific [4,15].

In cecal volvulus, in addition to the coffee bean sign, a markedly dilated cecum, a single air-fluid level, and absence of distal gas may also be observed. However, in 30% of cases, no findings are present. Some studies have reported that the presence of haustral markings is distinctive of cecal volvulus, which is usually absent in sigmoid volvulus; however, its sensitivity is very low, making it an unreliable marker [18].

In patients with transverse colon or splenic flexure volvulus, X-ray sensitivity is lower, and the diagnosis is usually made intraoperatively. When the radiograph shows suggestive findings, an abrupt change in caliber may be observed at the splenic flexure with the descending colon and sigmoid colon empty, or a loop of bowel may be seen folded in the upper abdomen. Furthermore, patients who have experienced multiple colonic volvuli may not exhibit the coffee bean sign in a new episode; instead, the radiograph may show diffuse colonic dilation [13]. 

Radiography not only allows confirmation of the volvulus diagnosis, it also allows the detection of complications like ischemia and intestinal perforation by recognising findings such as pneumoperitoneum or pneumatosis [6,13].

In children, abdominal X-rays are often nonspecific and are less useful in distinguishing volvulus from other disorders [11]. The sensitivity of the coffee bean sign for sigmoid volvulus in children is reported to be only 16 to 29%; therefore, additional imaging modalities may be required [10].

Findings on fluoroscopy

In situations where axial imaging fails to establish a definitive diagnosis, a water-soluble contrast enema can be useful, as it allows visualization of the "bird's beak” sign at the point of obstruction in 78% of cases of sigmoid volvulus and 44% of cases of cecal volvulus [7,8]. This sign characterizes the cutoff of the twisted bowel, demonstrating rapid spiral tapering of the colon at the site of obstruction resembling a bird's beak, also seen on CT [3,9,12].

The combination of plain abdominal radiographs and contrast enema images further increases the diagnostic rate, and the point of torsion can be identified in ≈70% of cases in adults [7]. In pediatric patients a plain abdominal X-ray is suggestive of sigmoid volvulus in 29% of cases, while combination with enema increases radiographic diagnostic sensitivity in 71-82% [10,11].

When using a contrast enema, a water-soluble contrast is much preferred over barium contrast, because the latter could cause a chemical peritonitis in the setting of a perforated colon [8]. Installation of contrast should stop as soon as the dilated colon is reached to reduce the chance of colonic perforation [9]. When perforation is suspected, an enema is strictly contraindicated [8].

Findings on computed tomography

CT demonstrates a sensitivity approaching 100% and a specificity greater than 90% for the diagnosis of volvulus [7,17]. Similar to plain radiography, diagnostic sensitivity is higher for sigmoid volvulus and tends to decrease when the volvulus involves other segments of the colon [13]. CT imaging can reveal multiple characteristic findings, including the coffee bean sign, bird’s beak sign, absence of rectal gas, abnormal colonic dilatation, whirl sign, transition point, and X-marks-the-spot sign. Other reported signs are less consistently reliable, such as the northern exposure sign, ileocolic vascular curvature, midline position of the appendix, steelpan sign, ileocecal twist, transverse colon decompression, distal colon decompression, air-fluid levels, air-feces levels, and cecal wall thinning [1,2,6,7,16-19].

Among these findings, the most reliable CT indicators for diagnosing sigmoid volvulus include a transition point within the sigmoid colon, marked dilatation of the sigmoid colon, absence of rectal gas, the whirl sign, coffee bean sign, bird’s beak sign, and the X-marks-the-spot sign [1,2,6,7,17,18]. In contrast, the CT findings most strongly associated with cecal volvulus are the coffee bean sign, transition point, whirl sign, and bird’s beak sign. Focusing on identifying these key findings may be more practical, efficient, and diagnostically useful than attempting to recognize every sign described in the literature [19]. Descriptions of the principal imaging signs are summarized in Table 2; however, the coffee bean sign and bird’s beak sign are not repeated, as they were discussed in earlier sections.

**Table 2:** Main signs of colonic volvulus on CT As described in previous sections, the coffee bean sign and bird's beak sign can also be seen on CT.

Sign	Description
The whirl sign	Indicates the twisting of the mesentery, the appearance of spiralated loops often shows the point of obstruction [14].
Transition point	Abrupt transition between distended and normal bowel [19]. The number of transition points on CT can also be predictive of the volvulus subtype: two for mesentero-axial and one for organoaxial [20].
X-marks-the-spot sign	is the presence of two crossing sigmoid transition points projecting from a single location in the coronal plane, showing an “X” created by the proximal and distal transition zones. This sign manifests in the advanced stages of intestinal rotation [14,17].

Other less visible signs are the steel pan and the split wall signs. The steel pan sign refers to the dilated colonic loops observed in cross-section, resembling the Trinbagonian percussion instrument made from a 55-gallon drum [21-23]. The split wall sign is characterized by visible mesenteric fat between the intestinal walls of a twisted loop, secondary to incomplete twisting or folding of the bowel loop. It is not sensitive, but has 100% specificity for the diagnosis of colonic volvulus. It is generally considered a marker of a less severe intestinal twisting [14,24].

Furthermore, CT allows evaluation of complications such as intestinal ischemia, in which pneumatosis, pneumoperitoneum, or submucosal edema are observed [6,8,13,18,19]. The “dark torsion knot sign” is a newly defined CT imaging factor of ischemia. It shows a sudden loss of mucosal enhancement in the volvulus torsion knot. This CT factor and a clinical factor (sepsis) were the only factors able to predict complicated volvulus sigmoid necessitating emergent surgery instead of colonoscopic detorsion as a primary treatment of choice [25].

CT has many other benefits, as it allows exclusion of other causes of obstruction due to a neoplasm or pseudo-obstruction, and it may help predict clinical outcome [6,8,13,18,19]. In a retrospective study published in 2025, Moloney et al. investigated whether CT findings in sigmoid volvulus are associated with clinical outcomes, including recurrence, appropriate management, and mortality. Eighty patients were evaluated between January 2007 and December 2021 and followed for at least six months. The main finding was that the risk of recurrence is associated with imaging-documented luminal distension ≥9 cm. This information can help determine whether a patient requires a selective sigmoidectomy. Furthermore, the direction of volvulus rotation was not associated with mortality, readmission, or recurrence risk [26].

Findings on magnetic resonance imaging

Magnetic resonance imaging (MRI) is mainly described to assess the suspicion of bowel obstruction or investigate causes of abdominal pain in vulnerable populations that must avoid ionizing radiation, such as pregnant women, fetuses, and neonates [8,27]. Even though midgut volvulus in the prenatal period is not going to be discussed in this paper, it's important to remark that the whirl sign and coffee bean sign still remain as the principal findings to suspect volvulus with MRI [28].

The imaging findings to realize the diagnosis of colonic volvulus based on MRI aren't different than the ones already described for CT: the coffee bean sign, the U-shaped distended sigmoid loops, the northern exposure sign, mesenteric whirl pattern, dilatation of the colon, intestinal air-fluid levels, transition points, and the split wall sign [27,29].

Findings on ultrasound

Another study technique in patients who cannot be exposed to ionizing radiation is ultrasonography (US). There is a large amount of information about US as the main study technique to diagnose midgut volvulus in pediatric patients, with a great sensitivity and specificity with the whirlpool sign, but there is little information about US in adults to diagnose volvulus because in this population X-ray and CT are the main techniques [7,8,30].

The signs that can be found in US are the ones previously described for other methods: the whirl sign, transition point, superior mesenteric artery cutoff, and bowel loop dilatation [27,30]. It is important to remark that the whirlpool sign in pediatric patients may be strikingly similar to the “target sign” or “donut sign” seen in intussusception due to concentric layers of echogenic bowel [31].

US has many advantages, including its portability to the bedside and absence of ionizing radiation; it is faster to perform compared with a CT or fluoroscopy and is also helpful to exclude other causes of acute abdominal pain, such as gallstones or kidney disease [27,32]. The main disadvantage is that it requires a well-prepared and experienced operator and it is limited by air interface, which may interfere with the visibility of the intra-abdominal structures [32].

Are the coffee bean sign and whirl sign exclusive to volvulus?

While the coffee bean sign suggests intestinal obstruction due to volvulus, it is not the only condition that can present with this finding. An ileosigmoid knot (IKS) can also show this image on a radiograph. CT is the gold standard for evaluating acute abdominal pain, where a double whirl sign may be revealed, implying that both the superior and inferior mesenteric arteries are involved, which is a characteristic feature of the ileosigmoid knot and not of volvulus. On CT, the double whirl can be distinguished because, in axial and sagittal images, the whirl sign extends from the lower border of L2 to the upper border of L5, a distinctive feature of the IKS, whereas in isolated volvulus, the whirl is limited to fewer slices. The importance of differentiating between obstructive pathologies lies in the fact that management may differ, since endoscopic resolution is not recommended for ileosigmoid knots, unlike in some cases of volvulus [23,26].

Discussion

Colonic volvulus represents an important cause of large bowel obstruction, and timely diagnosis is essential to prevent complications such as ischemia, perforation, and sepsis. Imaging plays a central role in the diagnostic process, with several modalities contributing complementary information. Although numerous radiologic signs have been described in the literature, their diagnostic value varies considerably depending on the imaging modality, the location of the volvulus, and the clinical context.

Another important consideration highlighted in the literature is the heterogeneity between studies reporting imaging findings. Many publications are retrospective case series or small cohort studies, which may introduce selection bias and variability in reported diagnostic accuracy. Furthermore, the frequency of specific radiologic signs may differ depending on the location of the volvulus - sigmoid versus cecal - and the stage of disease progression at the time of imaging. These factors should be taken into account when interpreting sensitivity values or attempting to generalize results across different clinical settings.

From a clinical decision-making perspective, imaging findings must be interpreted within the broader context of patient presentation and management pathways. In many institutions, the diagnostic approach begins with plain abdominal radiography due to its accessibility, followed by CT when findings are inconclusive or when complications are suspected. CT not only confirms the diagnosis but also helps determine the presence of ischemia or perforation, which directly influences treatment decisions such as endoscopic detorsion versus urgent surgical intervention. For this reason, CT plays a central role in modern diagnostic algorithms for colonic volvulus.

Despite the large number of radiologic signs described in the literature, focusing on a limited set of highly characteristic findings, such as the whirl sign, bird’s beak sign, transition point, and marked colonic dilatation, may be more clinically useful than attempting to identify every reported sign. Emphasizing these core features may improve diagnostic efficiency and facilitate earlier recognition of volvulus in emergency settings.

MRI and US have more limited roles in the evaluation of colonic volvulus but may be useful in specific clinical scenarios. MRI offers the advantage of avoiding ionizing radiation and may be considered in pregnant patients or when repeated imaging is necessary. However, its use in acute abdominal emergencies remains limited due to longer acquisition times and restricted availability in many institutions. Similarly, ultrasound can demonstrate signs such as the whirl pattern of twisted mesenteric vessels, particularly in pediatric or neonatal patients, but its diagnostic utility in adults is limited by bowel gas and operator dependency. Table 3 helps summarise the main characteristics, advantages, and disadvantages of each imaging method.

**Table 3:** Summary table of imaging methods and the main characteristics.

characteristics	X-ray	Fluoroscopy	CT	MRI	US
Signs	Coffee bean sign; northern exposure sign; dilated bowel loops; air–fluid levels; absence of distal gas [4,14,15,17]	Bird's beak sign [7,8]	Transition point; marked colonic dilatation; whirl sign; coffee bean sign; bird’s beak sign; X-marks-the-spot sign [1,2,6,7,17,18 ]	Coffee bean sign; mesenteric whirl pattern; dilatation of the colon; intestinal air-fluid levels; transition points; the split wall sign [27,29]	Whirl sign; transition point; superior mesenteric artery cutoff; bowel loop dilatation [27,30]
Sensitivity	26–42% (children 16–29%) [10]	~82% when combined with X-ray [10,11]	100% [7,17]	Limited data	Limited data
Advantage	Rapid; widely available; inexpensive; well tolerated [6,7,13]	Widely available; may provide diagnostic confirmation [8]	Gold standard in adults; high diagnostic accuracy [7,8]	No ionizing radiation; useful in pregnancy [27]	No ionizing radiation; bedside evaluation; useful in pregnancy and neonates [27]
Disadvantage	Ionizing radiation [7,8]	Ionizing radiation [7,8]	Ionizing radiation exposure [7,8]	Expensive; limited availability	Operator dependent; limited by bowel gas; requires patient preparation [32]
Clinical considerations	Findings may be nonspecific in children [10]	When perforation is suspected, an enema is strictly contraindicated [8]	Colonic dilatation >9 cm may predict recurrence [26]	Most evidence derived from prenatal imaging; limited adult data	Most literature describes pediatric use; limited adult data

Limitations

This narrative review has several limitations. Not all studies were published within the last 10 years; the selected literature varies in design, sample size, and results; the last two study techniques (MRI and US) have little to no information in adult patients; and only articles in Spanish and English were included.

## Conclusions

Colonic volvulus is an important cause of large bowel obstruction and requires prompt diagnosis to prevent complications such as ischemia and perforation. Although the coffee bean sign remains the most widely recognized radiographic finding, multiple additional radiologic signs have been described across different imaging modalities. Among imaging techniques, CT plays a central role in modern diagnosis, as it allows visualization of mesenteric torsion and associated complications. CT findings such as the whirl sign, bird’s beak sign, and transition point provide important diagnostic clues and should be recognized by clinicians and radiologists.

Regarding other techniques, contrast enema shows the bird's beak sign, which increases the sensitivity if combined with radiographs and is highly described for the diagnosis of volvulus in pediatric patients. When a patient must avoid ionizing radiation (like pregnant women or neonates), US and MRI are great options. A comprehensive understanding of these radiologic signs and the role of each imaging method may improve diagnostic accuracy and facilitate timely management of patients with suspected colonic volvulus.
